# Spatial distribution of intimate partner violence in Jordan: results from the 2023 Population and Family Health Survey

**DOI:** 10.3389/fpubh.2026.1734407

**Published:** 2026-05-15

**Authors:** Masood Ali Shaikh

**Affiliations:** Department of Medicine, College of Medicine, Korea University, Seoul, Republic of Korea

**Keywords:** intimate partner violence, women, spatial analysis, clustering, Jordan

## Abstract

**Background:**

Public health burden and human rights violations converge in intimate partner violence (IPV), reflecting sociocultural inequalities that deny women their basic right to safety.

**Methods:**

A secondary analysis of data from the 2023 Jordan Population and Family Health Survey (JPFHS) was conducted to analyze the geographic distribution of IPV in Jordan.

**Results:**

IPV proportions varied from 0 to 100% at the cluster/enumeration area (EA) level. Using inverse distance conceptualization, Moran’s *I* results (Index value: 0.051927; Z-score: 9.041519; *p* < 0.001) showed statistically significant clustering. Higher proportions were found in the northeastern governorates of Ajloun, Jarash, and Balqa, as well as in the northwestern areas of Zarqa and Amman governorates. Lower IPV proportions were observed in the Tafiela and Irbid governorates, as well as in the northeastern part of the Mafraq governorate; hot spot and cold spot analyses reinforced these findings. Cluster analysis using SaTScan revealed one primary and three secondary clusters of IPV. The spatial window of the primary cluster was located in an area encompassing the governorates of Ajloun, Jarash, Balqa, Zarqa, and Amman, with a relative risk (RR) of 1.78, a log-likelihood ratio (LLR) of 50.88, and a *p*-value of 0.001.

**Conclusion:**

Spatial clustering patterns, along with the identification of areas with high and low IPV prevalence, suggest the need for targeted resource allocation and interventions in Jordan to reduce and prevent the avoidable suffering and human rights violations experienced by married women.

## Introduction

Intimate partner violence (IPV) against women is a critical public health and human rights issue globally, affecting more than one-quarter of women (1). It is defined as a behavior within an intimate relationship that causes physical, sexual, and/or psychological harm to women (1). Globally, more than one-quarter (27%) of women aged 15–49 years who have been in intimate relationships report experiencing some form of physical and/or sexual violence committed by their partners (1). Several studies have indicated that IPV plays a significant role in the avoidable global health burden, contributing to physical harm, mental health challenges, reproductive health complications, and even deaths, while also imposing considerable economic costs on societies (2–7). In addition, IPV has been associated with intergenerational adverse impacts, as children who witness violence at home are at a higher risk of experiencing or perpetrating violence themselves in adulthood (8, 9).

Place, together with person- and time-related characteristics, is one of the three important dimensions in the study of descriptive and analytical epidemiology. The distribution of diseases and public health problems varies across geographical space, and the IPV burden is no exception. IPV prevalence varies across space due to location-specific sociocultural norms, practices, and the level of acceptance of women’s rights. Several studies have described the spatial distribution of IPV and have reported significant differences in the proportions within countries (10–15).

Across the Arab region, IPV is a widespread public health concern, with marked variation in the prevalence of its three types. A systematic review reported that, in the Arab world, sexual IPV affects between 3 and 40% of women, emotional IPV between 5 and 40%, and physical IPV between 6 and 59% (16). However, this systematic review also noted that differences in research methodologies and definitions of violence, particularly for emotional IPV, make direct comparisons across studies challenging. Studies on contextual factors related to IPV in Jordan and the wider Arab region have identified several associated determinants, including patriarchal family structures, intergenerational transmission of violence, women’s acceptance of violence as normal, extended family dynamics, socioeconomic disadvantage, lower educational attainment, and urban–rural differences in social norms. These factors are likely to vary geographically within the country and may contribute to spatial variation in the prevalence of IPV (17–25).

The Hashemite Kingdom of Jordan is located in the Middle East, where IPV represents a significant public health and human rights concern. It is primarily influenced by deep-rooted gender norms and cultural expectations that hinder the effectiveness of intervention efforts and support services (26–28).

The Demographic and Health Survey (DHS), implemented by the DHS Program, uses a standardized methodology to collect representative data in more than 90 countries, including Jordan. In Jordan, as in many other countries, the DHS (referred to in Jordan as the Jordan Population and Family Health Survey) remains the only source of nationally and subnationally representative data on various health and population indices over time (29). The 2017–18 Jordan Population and Family Health Survey (JPFHS) reported a 25.9% prevalence of IPV among ever-married women aged 15–49 years, perpetrated by their current or most recent husband (30), compared with the 31.7% prevalence reported in the 2012 JPFHS (30).

A critical first step in addressing subnational contributors to IPV is identifying subnational disparities. The spatial distribution of IPV has not been studied in Jordan. This study aimed to address this gap by analyzing the geographic distribution and disparities of IPV using data from the most recent Jordan Population and Family Health Survey. Using spatial analytical techniques, this study aimed to identify spatial patterns of lifetime IPV against women inflicted by their current or most recent husband in Jordan. Specifically, it aimed to map how IPV prevalence varies across the country, highlighting clusters and hot spots that may guide spatially targeted interventions.

## Methods

The geography of IPV in terms of its distribution, clustering, and hot spots in Jordan was analyzed, as described in the following sections. Details regarding the ethical approval procedures for the DHS are available at: https://dhsprogram.com/Methodology/Protecting-the-Privacy-of-DHS-Survey-Respondents.cfm.

### Study area and data source

The Hashemite Kingdom of Jordan is a middle-income country located in the Middle East and is administratively divided into 12 governorates. The population of Jordan was estimated at approximately 11.5 million in 2023, with more than 80% residing in the northwestern third of the country (31).

The 2023 Jordan Population and Family Health Survey (JPFHS 2023) was conducted by Jordan’s Department of Statistics, with technical support from ICF as part of the DHS Program, to provide nationally and subnationally (urban and rural; 12 governorates) representative estimates of population and health indicators. Data collection took place from 2 January to 15 June 2023. The survey used a stratified two-stage sampling design based on the 2015 Jordan Population and Housing Census. The stratification process involved dividing each governorate into urban and rural strata; subsequently, independent samples were drawn from each stratum. In total, 970 clusters were selected with probability proportional to each cluster’s size. In the second stage, 20 households were systematically selected from each cluster using equal probability.

The domestic violence (DV)_module included questions on IPV experiences and was administered to 50% of the sampled households. Within each household, one ever-married woman aged 15–49 years was randomly selected for the administration of the DV module. After obtaining verbal informed consent and ensuring privacy, the DV module was administered. In total, 5,495 ever-married women answered the IPV questions. The IPV questions inquired about specific instances of emotional, physical, and sexual violence experienced by the respondents. The term “husband” referred to the current spouse for married women and the most recent spouse for women who were divorced, separated, or widowed.

The GPS/geographic coordinates in the JPFHS 2023 were randomly displaced to ensure respondents’ confidentiality. For urban clusters, this adjustment displaced locations by up to 2 km, while rural clusters were displaced by up to 5 km, with 1% of these rural clusters displaced by up to 10 km. However, this displacement ensured that all clusters still remained within their respective district and governorate boundaries within the country.

Ethical approval for the 2023 Jordan Population and Family Health Survey (JPFHS 2023) was provided by the Jordanian Department of Statistics and the Institutional Review Board of ICF. The author was granted access to the data by ‘The DHS Program’^1^ through its online request form for secondary analysis of de-identified JPFHS 2023 data. The author did not collect any data. Full details of the JPFHS 2023, including survey methodology, sampling framework, questionnaires, and survey weight generation procedures, are freely available on the DHS Program website.

### Study variable

In the JPFHS 2023, questions were asked about three types of IPV: Emotional violence was assessed based on whether the current or most recent husband had ever: “*said or did something to humiliate her in front of others; threatened to hurt or harm her or someone she cared about; insulted her or made her feel bad about herself*”; *ignored or neglected her; ignored or neglected her sexually; threatened her or kicked her out of the house.”* Physical violence was assessed based on whether the current or most recent husband had ever: “*pushed her, shook her, or threw something at her; slapped her; twisted her arm or pulled her hair; punched her with his fist or with something that could hurt her; kicked her, dragged her, or beat her up; tried to choke her or burn her on purpose; attacked her with a knife, gun, or other weapon.*” Finally, sexual violence included experiences in which the current or most recent husband had ever: “*physically forced her to have sexual intercourse when she did not want to; physically forced her to perform any other sexual acts she did not want to; forced her with threats to perform sexual acts she did not want to.*” A binary composite variable labeled “IPV” was developed, where a value of “1″ was assigned to respondents who reported ever experiencing any type of emotional, physical, and/or sexual violence from their current or most recent husband. If none of these experiences were reported, the variable was coded as “0.”

### Data analysis

The de-identified data for the JPFHS 2023 were obtained through the Measure DHS website^2^ with prior authorization. To calculate weighted IPV prevalence at the enumeration area (EA) level in Jordan, STATA version 18 (Texas, USA) was used. For mapping and spatial analysis, ESRI’s ArcMap version 10.8.1 was utilized. Administrative boundary data for Jordan were obtained from the Spatial Data Repository of the DHS Program (32). The World Geodetic System 1984 (WGS 84) geographic coordinate system (GCS) was used for creating maps.

Weighted IPV proportions at the cluster level were calculated using Stata, exported to an Excel file, and then linked to the geographic coordinates of each cluster in ArcMap. Due to the unavailability of geographic coordinates for one cluster, it was excluded from the analysis, and 13 clusters were excluded because IPV information was not available. The spatial distribution of IPV proportions at the cluster level across the remaining 956 clusters, represented as percentages, was subsequently visualized on the map and spatially analyzed.

To meet the study’s aim of identifying spatial patterns of IPV across Jordan, three spatial methods were sequentially applied: First, to determine whether clustering exists; second, to identify where clusters occur; and third, to independently validate the identified clusters. To assess the presence of spatial autocorrelation/clustering in IPV proportions in Jordan, the Global Moran’s *I* statistic was applied. This statistic determines the extent to which similar values are spatially clustered across the study area. Moran’s *I* values range from +1 to −1, where positive values indicate clustering, negative values indicate dispersion, and values near zero indicate randomness; and a *p*-value of less than 0.05 was considered statistically significant. A local indicator of spatial clustering, the Getis-Ord Gi* statistic, was also used. This method compares the distribution of individual points with their neighboring points in terms of high-value points being surrounded by similarly high values and low-value points being surrounded by similarly low values. The Gi_Bin field provides z-scores and *p*-values for each point and identifies statistically significant points at the 90%, 95%, and 99% confidence levels that are clustering at both high and low values or hot and cold spots/points, respectively.

The optimized hot spot analysis tool in ArcMap version 10.8.1 (Spatial Analyst extension) was used to streamline this process and identify statistically significant clusters in the spatial data.

Finally, a spatial scan statistic was used to detect statistically significant clusters of high and low IPV prevalence. Within each enumeration area, women who reported ever experiencing IPV were considered cases, while those who did not were considered controls. Since the metric studied was binary (presence or absence of IPV), a Bernoulli model was applied using Kulldorff’s SaTScan software (version 10.2.5.) This method uses a circular scanning window that systematically moves across the study area to identify statistically significant clusters. To capture clusters of varying sizes, the maximum spatial cluster size was set to less than 50% of the population by default. Based on *p*-values and likelihood ratio tests, primary clusters were identified, with the cluster yielding the largest likelihood ratio indicating the most probable primary cluster.

These three spatial statistical methods are complementary: Moran’s *I* tests for overall clustering, followed by the Getis-Ord Gi* statistic, which identifies the locations of statistically significant hot and cold spots. Finally, Kulldorff’s SaTScan independently identifies statistically significant clusters using a scanning window approach and triangulates the identified patterns through the Getis-Ord Gi* analysis.

## Results

The JPFHS 2023 surveyed 5,495 eligible women regarding IPV. The lifetime prevalence of IPV perpetrated by current or most recent husbands among ever-married women aged 15–49 years was 18.28% (95% CI: 16.38–20.35%).

**Figure 1:**
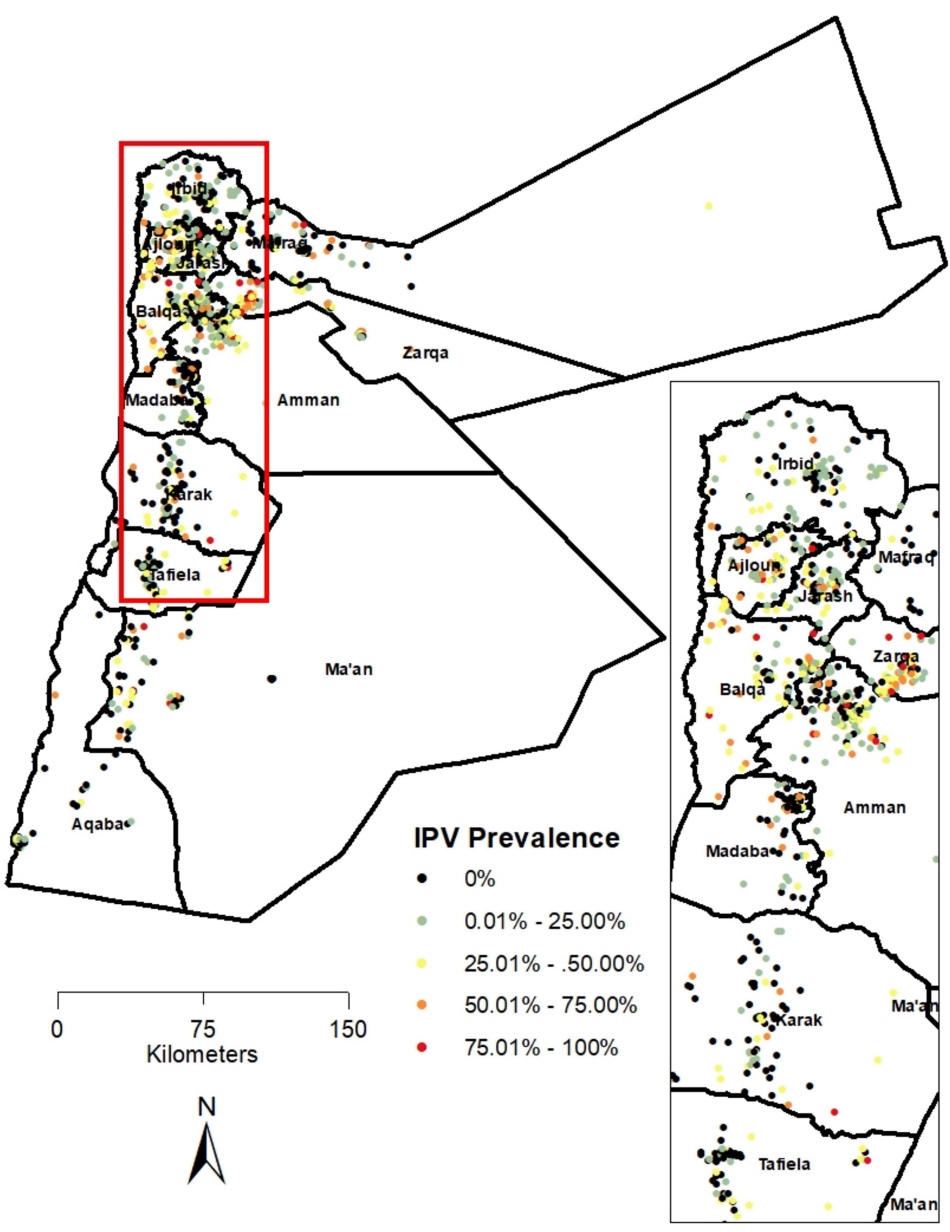
Spatial distribution of cluster points and proportions of IPV in Jordan (JPFHS 2023).

At the cluster/enumeration area level, the proportions of IPV ranged between 0 and 100%. This distribution is illustrated in Figure 1, which depicts the locations of the clusters/EAs along with their respective IPV proportions across various governorates. Northeastern governorates of Ajloun, Jarash, and Balqa showed higher IPV proportions, along with the northwestern areas of Zarqa and Amman governorates. In contrast, lower IPV proportions were found in the Tafiela and Irbid governorates, as well as in the northeastern part of the Mafraq governorate. Subsequently, these findings were confirmed by the hot spot analysis, which revealed statistically significant clusters of high and low values in these governorates, indicating the presence of “hot” spots and “cold” spots within these areas.

Moran’s *I* statistic was calculated to determine spatial autocorrelation using three spatial conceptualizations with row standardization: Zone of indifference, inverse distance, and inverse distance squared. Only the inverse distance approach produced a statistically significant Moran’s *I* result (Index value: 0.051927; Z-score: 9.041519; *p* < 0.001), indicating positive but weak spatial autocorrelation that followed the distance decay model, in which the influence between points decreases gradually with distance rather than abruptly.

**Figure 2:**
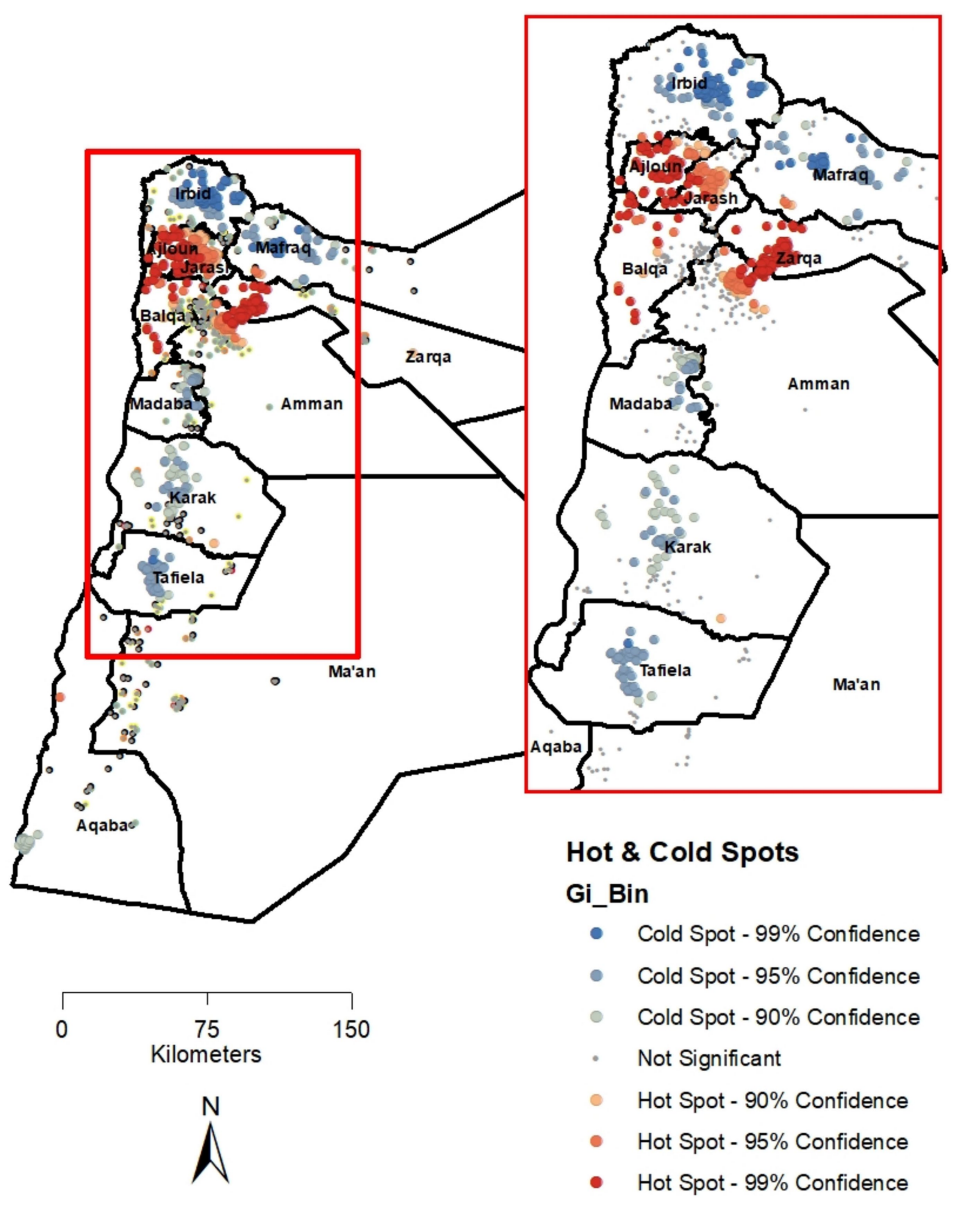
Results of the IPV hot spot analysis based on the Getis-Ord (Gi*) statistic, Jordan (JPFHS 2023).

The Getis-Ord Gi* statistic was computed to identify statistically significant hot and cold spots of IPV prevalence in the country. Figure 2 shows these results and confirms the pattern seen in Figure 1. Hot spots were limited to the northwestern governorates of Ajloun, Jarash, and Balqa, as well as the western areas of the Zarqa and Amman governorates. In contrast, cold spots were identified in the Tafiela, Irbid, and Mafraq governorates.

**Figure 3:**
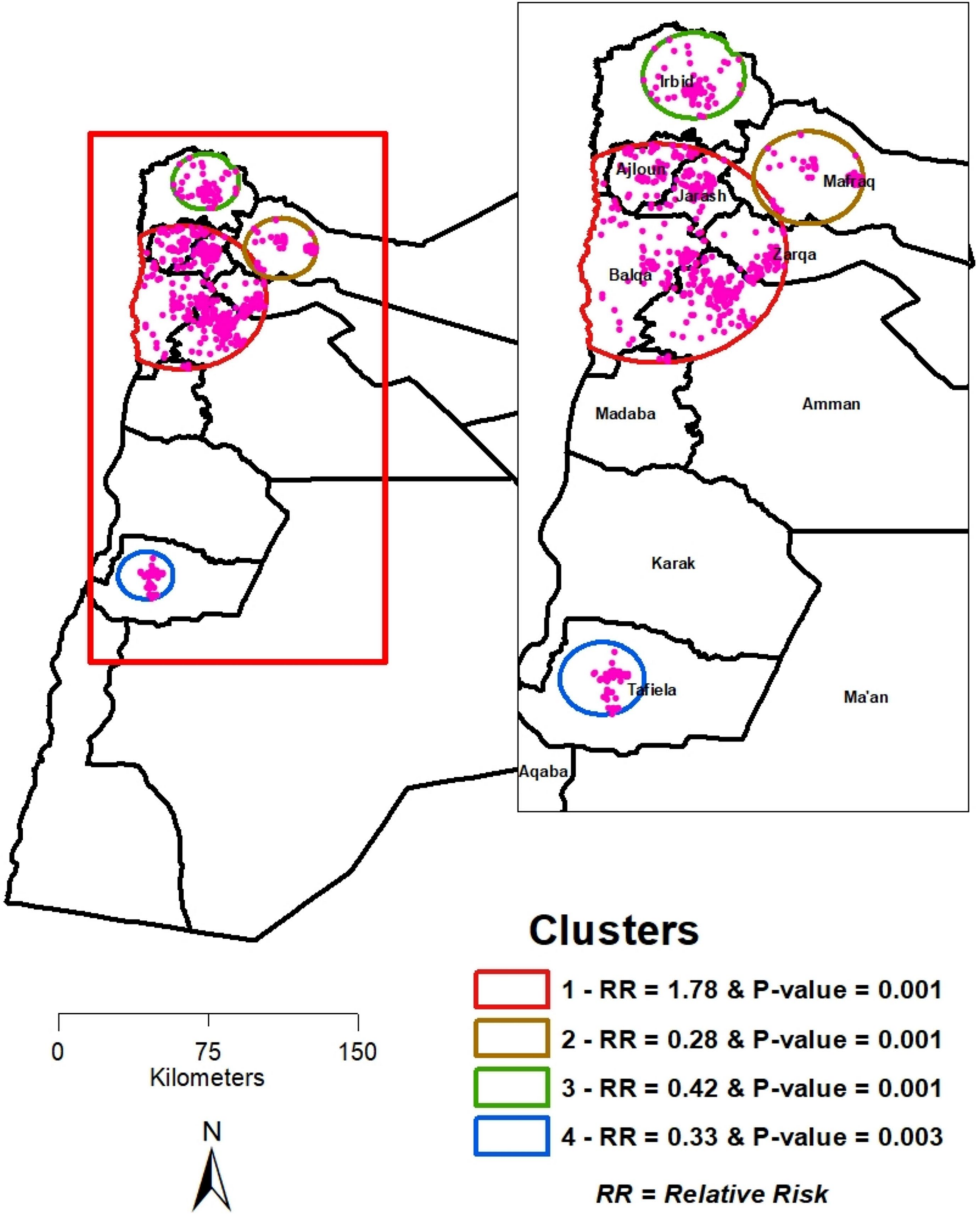
Clusters of IPV proportions in Jordan identified using SaTScan analysis (JPFHS 2023).

Figure 3 shows the results of the SaTScan analysis, identifying one primary and three secondary clusters of IPV. The spatial window of the primary cluster was located in the area surrounding the governorates of Ajloun, Jarash, Balqa, Zarqa, and Amman, with a relative risk (RR) of 1.78, a log-likelihood ratio (LLR) of 50.88, and a *p*-value of 0.001. This indicates that women within this spatial window had a 1.78 times higher risk of ever experiencing IPV compared with women outside this window.

The three secondary clusters revealed lower IPV proportions. The second cluster’s spatial window was situated in the Mafraq governorate, with an RR of 0.28 (LLR: 29.25; *p* < 0.001), indicating that women within this spatial window had a 28% risk of IPV compared with women outside the window. The third cluster’s spatial window was situated in the Irbid governorate, with an RR of 0.42 (LLR: 24.33; *p* < 0.001), indicating that women within this spatial window had a 42% risk of IPV compared with women outside the window. The fourth cluster’s spatial window was situated in the Tafiela governorate, with an RR of 0.33 (LLR: 13.73; *p* < 0.003), indicating that women within this spatial window had a 33% risk of IPV compared with women outside the window.

## Discussion

This is the first study to report on the geography of intimate partner violence in Jordan. The most recent spatial data from the nationally and sub-nationally representative 2023 Jordan Population and Family Health Survey were used. There was substantial variation in IPV prevalence across the country, with higher proportions observed in the governorates of Ajloun, Jarash, and Balqa, as well as in the northwestern areas of the Zarqa and Amman governorates, while lower IPV proportions were found in the governorates of Irbid, Mafraq, and Tafiela.

The statistically significant result of Moran’s *I*, using the inverse distance conceptualization, suggests that spatial clustering in the data follows a continuous distance decay effect, in which points exert influence on one another in a gradual manner that diminishes with increasing distance. Moran’s *I* is a global statistic and assesses overall spatial patterns. In contrast, hot and cold spot analyses, such as the Getis-Ord Gi* statistic, are local statistics that identify significant hot and cold spots in the study area of high or low values. The Global Moran’s *I* was statistically significant, but the overall global spatial autocorrelation was weak. However, the local hot spot analysis showed more meaningful localized spatial clustering.

Both metrics were found to be statistically significant using the most recent data in Jordan. Kulldorff’s SaTScan analysis substantiated the visualization of IPV prevalence shown in Figure 1 and reinforced the results of the hot and cold spot analyses, underscoring the need for geographically targeted responses to curb and ultimately eradicate IPV in Jordan. IPV was not randomly distributed across the country but instead exhibited distinct clusters of high and low concentrations. The statistically significant primary cluster of high IPV prevalence across the five governorates highlights the need for policies and interventions to identify and address the factors that are harming women through IPV. Spatial cluster identification is an important descriptive step that can generate hypotheses regarding where response might be needed. However, placing these identified patterns into geographically targeted interventions requires a nuanced understanding of the underlying mechanisms shaping the observed clustering. This mechanism includes local socioeconomic conditions, cultural norms, and access to services.

The cross-sectional design of the DHS allows for the examination of correlates of IPV but does not permit causal inferences. The observed spatial clustering of IPV in the northwestern governorates of Ajloun, Jarash, and Balqa, as well as parts of Zarqa and Amman, may hypothetically reflect geographic variation in contextual factors identified in Jordan-specific literature. Collectively, previous studies have recommended that differences in patriarchal norms, family structures, socioeconomic conditions, and community attitudes across Jordan’s governorates likely underlie the spatial patterns observed in this study (17–25).

As there are no previous studies on the spatial analysis of IPV in Jordan, direct comparisons or contrasts with existing findings are not possible. Moreover, the most recent DHS in Arabic-speaking countries such as Egypt, Morocco, Tunisia, and Yemen was conducted between 1988 and 2014 (29). However, geographically varying prevalence of IPV within countries are well documented. Studies from several African countries (11–15) and one Asian country (10) have clearly shown that geography plays a significant role in the distribution of the lifetime prevalence of IPV perpetrated against women by their current or most recent husband/partner, using DHS data. Consistent with these reports, a study from 10 West African countries also reported similar geographical variations in IPV (33). In a similar vein, studies on IPV prevalence within the past 12 months using DHS data have also reported geographical disparities (34–36). Although spatial analyses of IPV from Sub-Saharan African and other countries are referenced for their methodological comparability, particularly in their use of DHS data, the sociocultural and economic contexts of these countries may differ from those of Jordan.

Several studies on the spatial analysis of IPV using point data have also employed kriging to interpolate IPV prevalence in areas where data were not collected (10, 13, 15, 34). In the JPFHS 2023 dataset, points are concentrated almost exclusively on the eastern side, reflecting the country’s population distribution, while the central and western regions essentially lack enumeration areas. This distribution of enumeration areas would likely produce misleading interpolation results, as the absence of population in the central and eastern regions does not represent meaningful population-based estimates.

The primary strength of this study lies in the use of the most recent and nationally representative data on IPV. However, the use and results of cross-sectional survey data need to be interpreted with caution. IPV was self-reported by women and could be influenced by social desirability bias, feelings of shame, and the inherent stigma associated with this personal issue, particularly against the backdrop of cultural norms. This reluctance to disclose IPV is unlikely to have a uniform geographic distribution.

However, a major limitation of this study is that only ever-married women were studied regarding their IPV experiences with their current or most recent husband, thereby excluding premarital and extramarital IPV experiences. In addition, never-married women and women in lesbian relationships were not included, nor were female partners of respondents in bisexual relationships, in the IPV assessment in Jordan. Collectively, these limitations may have led to an underestimation of the true burden of IPV in the country.

As the DV module was administered to only half of the sampled households and to one woman per household, there were few respondents per cluster contributing to each cluster’s IPV proportion. As a result, a difference of even one or two reported cases can change a cluster’s proportion from 0 to 100%. However, this variation is a feature of the DHS design rather than necessarily reflecting true geographic differences. However, the hot spots and cold spots identified in this study cover entire governorates across multiple clusters, which makes it less likely that the overall spatial patterns are simply the result of small numbers within individual clusters. Furthermore, the spatial analytical methods used—namely, Moran’s *I*, the Getis-Ord Gi* statistic, and Kulldorff’s SaTScan—evaluate whether clusters with similar values are spatially concentrated and apply statistical significance testing. The Getis-Ord Gi* statistic identifies a hot or cold spot only when a cluster and its neighbors collectively show significantly high or low values, and SaTScan aggregates observations within a scanning window and applies a likelihood ratio test. Therefore, these properties reduce, but do not completely remove, the potential influence of survey design-induced variability on the detection of spatial patterns.

At the cluster level, IPV proportions in clusters with a small number of respondents may be sensitive to minor variations in the number of IPV cases. The Getis-Ord Gi* statistic and Kulldorff’s SaTScan inherently mitigate the influence of isolated extreme values by evaluating spatial patterns across neighboring clusters when applying statistical significance testing. However, the possibility of variance instability in individual cluster-level proportions remains. Another avenue for future studies would be the application of model-based small area estimation using Bayesian spatial models to yield more stable subnational estimates.

Another limitation is that the use of a composite IPV metric does not differentiate between the three individual types of IPV. Future studies on the spatial distribution of each IPV type would provide a more nuanced understanding of IPV geography to help inform protection measures for affected women in terms of specialized legal and healthcare services, as well as emergency shelter planning and location placement. However, DHS clusters are the result of a stratified survey sampling design and may not necessarily correspond to all intervention-relevant neighborhoods and communities. Furthermore, the deliberate displacement of cluster GPS coordinates to protect respondent anonymity introduces a degree of spatial inaccuracy. However, the spatial patterns identified in this secondary analysis span entire governorates rather than individual clusters, which may mitigate the influences of cluster-level instability and GPS displacement on the overall findings. In addition, the composite measure of IPV does not capture the frequency and severity of IPV. Finally, the use of lifetime IPV experience, mapped to the respondent’s current location, may not necessarily reflect the place where the violence actually occurred.

The hallmarks of descriptive epidemiology involve studying characteristics in terms of time, place, and person. This study was limited to the spatial analysis of IPV in the country. Future studies on the geography of IPV need to incorporate additional dimensions, such as women’s beliefs regarding the acceptability of IPV and awareness of IPV perpetrated by fathers against mothers, to better capture the influence of IPV correlates within a spatial context. In addition, future studies need to spatially analyze the relationship between IPV and explanatory factors using geographically weighted regression (GWR), which models how the influence of explanatory variables varies across geographic space.

As the DHS is cross-sectional in nature, the observed spatial patterns of lifetime IPV in ever-married women should not be interpreted as stable place-based disparities. Determining IPV causality in terms of risk factors would require longitudinal studies. The identified clusters and hot spots may help guide geographically targeted interventions and inform resource allocation decisions, supplemented and corroborated by future studies. Understanding the geography of IPV may help improve women’s health and human rights in Jordan by supporting spatially targeted IPV interventions, including health education and promotion campaigns aimed at ensuring women’s safety and protection.

## Data Availability

The data analyzed in this study is subject to the following licenses/restrictions: the author was granted permission and provided access to the data by the ‘The DHS Program’ (https://dhsprogram.com) through its online request form for secondary analysis of the deidentified JPFHS 2023 data. Author did not collect any data. Requests to access these datasets should be directed to ‘The DHS Program’ (https://dhsprogram.com).

## Footnotes

1
https://dhsprogram.com

2
https://dhsprogram.com/Data/
